# ConFormer-Net: Spatiotemporal Modeling for Landslide Detection Using Multi-Temporal SAR Data

**DOI:** 10.3390/s26185943

**Published:** 2026-09-19

**Authors:** Shaofei Lan, Daming Wu, Peng Lu, Beinan Guo, Zixiao Li

**Affiliations:** 1 National Institute of Natural Hazards, Ministry of Emergency Management of China, Beijing 100085, China; lanshaofei24@mails.ucas.ac.cn (S.L.); lupeng24@mails.ucas.ac.cn (P.L.); 202131260004@mail.bnu.edu.cn (B.G.); zixiaoli@ninhm.ac.cn (Z.L.); 2 Key Laboratory of Compound and Chained Natural Hazards Dynamics, Ministry of Emergency Management of China, Beijing 100085, China

**Keywords:** landslides, spatiotemporal modeling, CNN, transformer, SAR imagery

## Abstract

**Highlights:**

**What are the main findings?**

- ConFormer-Net combines multiscale dilated convolutions, sinusoidal positional encoding, Transformer-based temporal modeling, and cross-attention to jointly capture spatial structures and long-range temporal changes in multi-temporal SAR imagery.
- ConFormer-Net achieved an F1-score of 95.27%, an accuracy of 95.29%, a precision of 96.79%, and a recall of 93.79%, achieving the best overall performance among all comparison models; ablation experiments identified temporal modeling as the primary contributor to performance improvement.

**What are the implications of the main findings?**

- Joint spatiotemporal modeling improves the discrimination of landslide-induced changes from vegetation disturbance, terrain shadows, bare ground, and SAR speckle noise, thereby reducing false detections in complex mountainous environments.
- ConFormer-Net achieved high detection accuracy and stable performance under different terrain conditions and landslide morphologies, providing an effective approach for landslide detection in complex terrain environments.

**Abstract:**

Landslides are characterized by sudden occurrence, severe destructiveness, and widespread spatial distribution. Therefore, rapid and accurate landslide detection is essential for reducing infrastructure damage and safeguarding human lives. Conventional landslide monitoring methods are generally time-consuming, labor-intensive, and inefficient, while existing deep learning-based landslide detection methods remain limited in multiscale spatial structure representation, temporal sequence modeling, and spatiotemporal feature fusion. To address these limitations, this study proposes ConFormer-Net, a spatiotemporal landslide detection model that integrates convolutional neural networks (CNNs) with a Transformer architecture for landslide detection from multi-temporal synthetic aperture radar (SAR) imagery. The proposed model adopts a hybrid architecture and incorporates a cross-attention mechanism. Specifically, a dilated convolutional network is employed to extract local spatial features of landslides, while a Transformer encoding module models the temporal dependencies among multi-temporal SAR observations. The extracted spatial and temporal features are subsequently integrated through the cross-attention mechanism to achieve accurate landslide detection. The overall detection performance of ConFormer-Net was first evaluated using samples from different regions in the publicly available Sen12Landslides dataset. The proposed model was then compared with CNN, CNN-LSTM, ConvLSTM, GRU, CNN3D and ResNet50 models. The results demonstrate that ConFormer-Net achieved the best overall performance, with an F1-score of 95.27%, an accuracy of 95.29%, a precision of 96.79%, and a recall of 93.79%. These results indicate that ConFormer-Net enables highly accurate landslide detection while maintaining moderate model complexity, demonstrating its effectiveness for landslide detection from multi-temporal SAR imagery.

## 1. Introduction

Landslides refer to the downslope movement of unstable rock or soil masses along a shear failure surface under the influence of gravity, typically triggered by factors such as rainfall, earthquakes, and human activities [1]. They are characterized by long-term development, rapid triggering, and severe destructive potential. Once a landslide occurs, it can pose serious threats to infrastructure, property, and human life, while also causing substantial damage to ecosystems [2,3,4]. Owing to their sudden occurrence, destructive nature, and extensive impact, timely responses to landslide disasters remain challenging. Therefore, the dynamic detection of landslides and the early identification of potential hazards are of great significance for enabling targeted measures for disaster prevention, emergency response, and risk reduction [5].

To minimize the loss of life and property caused by natural hazards, extensive research has been conducted worldwide on landslide detection and early identification. Conventional landslide detection and identification methods, including manual ground observations, global positioning systems, and unmanned aerial vehicle surveys, are often constrained by difficulties in establishing monitoring sites, high operational costs, limited large-scale monitoring capacity, and susceptibility to weather conditions and cloud cover. These limitations make it difficult to ensure reliable monitoring accuracy and present considerable challenges for landslide detection over large areas, across multiple spatial scales, and at high temporal frequencies [6,7,8]. With the continuous development of remote sensing technology, satellite-based landslide monitoring has gradually become an important research focus. In particular, synthetic aperture radar (SAR), with its day-and-night, all-weather, and wide-area observation capabilities [9,10,11], effectively compensates for many limitations of conventional landslide monitoring methods [12,13,14] and has been widely applied to landslide deformation detection and early identification [15,16,17].

In recent years, deep learning has been widely applied to remote sensing information extraction because of its powerful feature extraction and representation capabilities, reducing the dependence of conventional machine learning methods on manually designed features [18,19,20,21,22]. In particular, convolutional neural networks (CNNs) can automatically learn local textures, morphological patterns, and structural information from remote sensing imagery and have achieved remarkable performance in remote sensing-based landslide detection tasks [23,24,25]. However, one limitation of CNNs is that the effective receptive field produced by stacking small convolution kernels is generally much smaller than the theoretical receptive field, thereby restricting their ability to model global contextual information and long-range dependencies [26,27,28]. In complex environments, this limitation may lead to semantic noise and the misclassification of objects with similar features. Consequently, CNN-based models may incorrectly identify vegetation, bare ground, terrain shadows, and other background objects with backscattering characteristics similar to those of landslides as landslide areas. Meanwhile, they may also struggle to comprehensively represent the multiscale morphological structures of landslides, including rear scarps, lateral boundaries, and depositional boundaries [29].

The occurrence of a landslide is often accompanied by changes in surface deformation, backscattering characteristics, and land-cover conditions. Therefore, relying solely on single-temporal imagery or local spatial features is insufficient to fully characterize the dynamic changes occurring before and after a landslide event. To address this issue, previous studies have introduced recurrent neural networks, including long short-term memory networks (LSTMs), bidirectional long short-term memory networks (Bi-LSTMs), and gated recurrent units (GRUs), to model the temporal evolution of landslides and predict landslide displacement [30,31,32]. For example, Zhu et al. [33] proposed a stacked LSTM model in which multiple hidden layers were used to model complex dependencies and hierarchical features, thereby improving landslide prediction performance. However, these recurrent neural networks explicitly represent temporal dependencies through the recursive updating of hidden states. This sequential computation mechanism limits the efficient parallel processing of long time series and consequently constrains their ability to model the long-term evolution and long-range temporal dependencies of landslides [34].

To overcome these limitations, the Transformer architecture was introduced. By using a self-attention mechanism, Transformers can capture global relationships among different time steps and efficiently model global temporal dependencies [35]. Previous studies have applied Transformers to landslide detection and demonstrated their effectiveness in this task [36,37]. Nevertheless, Transformers lack the local inductive biases inherent in convolutional operations and may therefore overlook fine-grained local textures and precise boundary information, particularly when detecting small and fragmented landslides [38]. In addition, the self-attention mechanism itself does not inherently encode positional information and is therefore unable to distinguish spatial or temporal order without additional positional representations. In multi-temporal SAR-based landslide detection, this limitation prevents the model from naturally distinguishing the chronological order of different observations, thereby restricting its ability to represent critical temporal differences and continuous evolutionary patterns before and after landslide occurrence [39].

A single CNN or Transformer architecture cannot simultaneously achieve detailed local spatial representation and effective long-range temporal dependency modeling for multi-temporal landslide detection. By combining the local feature extraction capability of convolutional networks with the global temporal modeling capability of Transformers, CNN–Transformer hybrid architectures have gradually become an important modeling strategy in remote sensing change detection and landslide identification [40]. However, existing CNN–Transformer methods still exhibit limitations in spatiotemporal feature fusion. Most existing fusion strategies rely on simple stacking, concatenation, or weighted summation, which cannot fully exploit the complementary advantages of CNN-derived local detail features and Transformer-derived long-range temporal dependency features. These strategies also lack sufficiently deep interaction modeling between spatially discriminative features and temporally evolving features [41]. When applied to landslide detection, such limitations may prevent the model from effectively distinguishing genuine landslide-induced disturbances from complex background changes, thereby reducing detection accuracy and stability. Therefore, a more refined spatiotemporal feature fusion mechanism is required to enhance the joint representation of local spatial characteristics and temporal change patterns associated with landslides [42].

To address the insufficient representation of local spatial details, the limited modeling of long-range temporal dependencies, and the inadequate interaction between spatial and temporal features in multi-temporal SAR-based landslide detection, this study proposes ConFormer-Net, a CNN–Transformer hybrid architecture designed for landslide detection from multi-temporal remote sensing imagery. First, a multiscale spatial feature extraction module is constructed using dilated convolutions. This module enlarges the receptive field while preserving spatial resolution, thereby enhancing the representation of local landslide morphology, including rear scarps, lateral cracks, and depositional boundaries. Subsequently, a Transformer-based temporal modeling module is introduced, together with positional encoding that incorporates the chronological order of multi-temporal observations. This enables the model to capture long-range temporal dependencies associated with surface changes before and after landslide occurrence. On this basis, a cross-attention mechanism is designed to adaptively fuse the local spatial features extracted by the CNNs with the long-range temporal dependency features modeled by the Transformer, thereby strengthening the interaction between spatially discriminative information and temporal evolution information. Through these designs, this study aims to improve the model’s ability to detect genuine landslide-induced disturbances under complex terrain and background conditions and to enhance the accuracy and stability of landslide detection using multi-temporal SAR data. The main contributions of this study are summarized as follows:

1. A spatiotemporal collaborative modeling framework is proposed for landslide detection using multi-temporal SAR data. Unlike conventional CNN-based methods that primarily focus on local spatial features, ConFormer-Net simultaneously captures local landslide morphology and long-term temporal changes, thereby improving the representation of landslide evolution in complex mountainous environments.
2. To address the inability of Transformers to inherently perceive positional information, sinusoidal positional encoding is introduced into the temporal modeling module. This encoding incorporates the temporal positions of individual observations and enables the self-attention mechanism to distinguish their chronological order. Consequently, the model can more effectively differentiate temporal changes before and after landslide occurrence and improve its representation of the temporal evolution of landslides.
3. A spatiotemporal feature fusion module based on cross-attention is designed. The local spatial features extracted by the CNNs are used as the Query, while the long-range temporal dependency features modeled by the Transformer are used as the Key and Value. This design enables adaptive interaction modeling between spatial information and long-range temporal dependencies, thereby enhancing the model’s ability to distinguish landslide areas from non-landslide areas under complex terrain conditions and background interference.

## 2. Materials and Methods

### 2.1. Landslide Sample Areas and Dataset

This study conducted experiments using the publicly available Sen12Landslides dataset released by Höhn et al. Sen12Landslides is a large-scale, multi-temporal remote sensing dataset developed for satellite-based landslide detection. It compiles landslide events from 15 regions worldwide between 2016 and 2023 and contains approximately 75,000 landslide annotations and more than 12,000 standardized image patches. The dataset reflects the spatial variability and temporal change characteristics of landslides under different geographical conditions. During the construction of the Sen12Landslides dataset, multi-temporal observation sequences were generated for individual landslide events. Landslide records and NDVI time-series changes were used by the dataset developers to determine or refine the event date and define the corresponding pre- and post-event temporal windows, after which a fixed number of remote sensing images were selected from the long-term observation series to form standardized samples. This temporal information was used only for dataset construction and was not included as input features for ConFormer-Net. In addition, the study areas were divided into regular grids of 128 × 128 pixels, with each grid serving as the basic unit for generating image patches. All samples were stored in NetCDF format. Grids containing landslides were assigned pixel-level binary labels, while non-landslide areas were randomly selected to maintain a relatively balanced class distribution [43].

**Figure 1:**
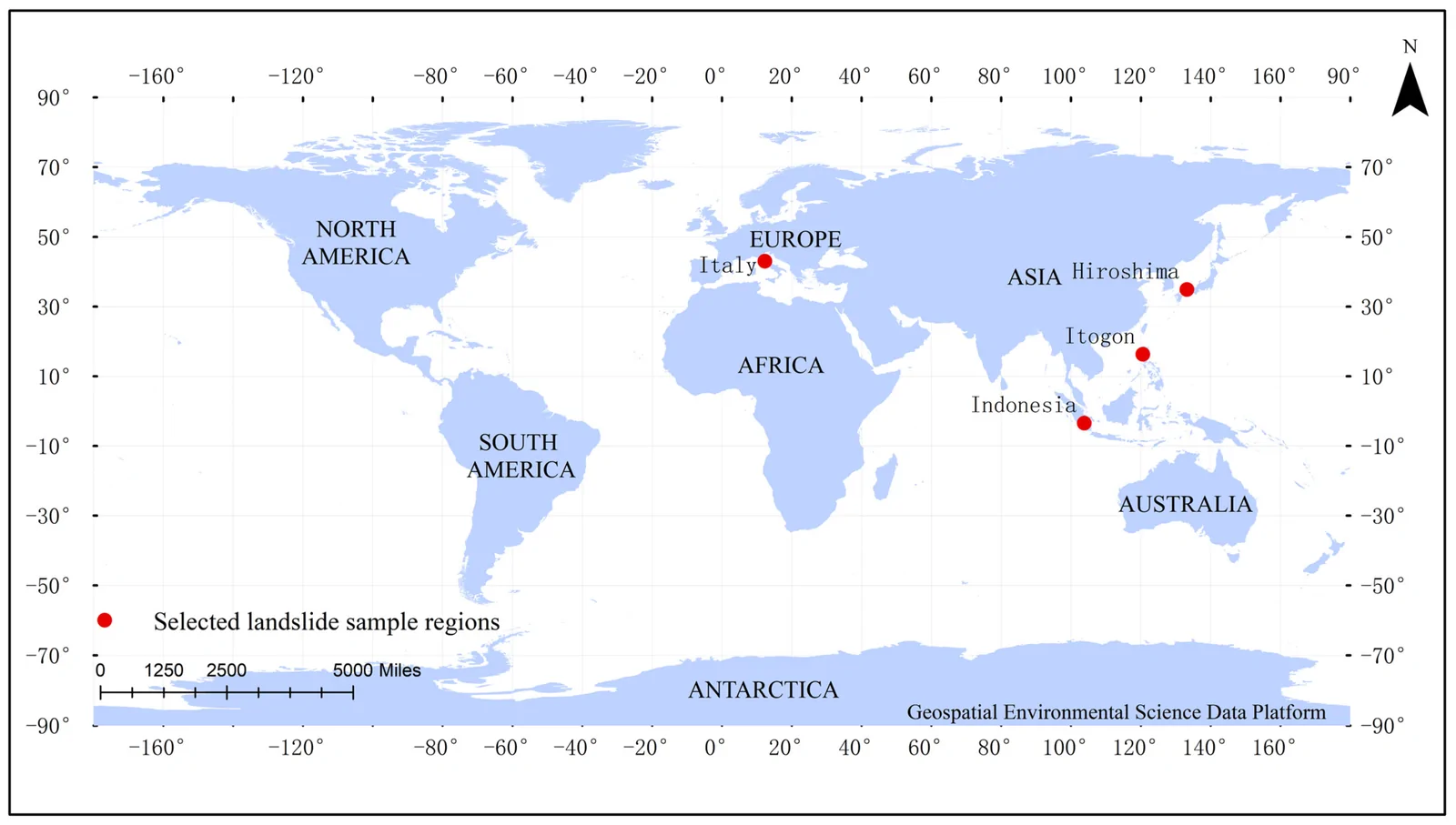
Spatial distribution map of landslide samples. The red dots indicate the four representative landslide sample regions selected from the Sen12Landslides dataset in this study: Hiroshima, Indonesia, Italy, and Itogon.

In this study, the harmonized Sentinel-1 ascending-orbit (S1-asc) data from the Sen12Landslides dataset were used in the experiments. Among the 15 regions included in the dataset, Hiroshima, Indonesia, Italy, and Itogon were selected to construct the experimental subset for model training and validation. The four selected regions are geographically distributed across different countries and areas, providing spatially diverse study sites. In addition, the proportions of landslide samples in the four regions range from 49.3% to 54.8%, resulting in a relatively balanced composition of landslide and non-landslide samples. Considering the spatial distribution of the study areas, sample composition, and the need to maintain a manageable experimental scale, these four regions were selected for the experiments. For data preparation, the corresponding S1-asc NetCDF files were extracted, and the VV- and VH-polarized SAR data were used as model inputs. The S1-asc data used in this study were the harmonized products provided by Sen12Landslides. During construction of the original dataset, the Sentinel-1 IW data were processed as Normalized Radar Backscatter (NRB) products with radiometric terrain correction, resampled to a spatial resolution of 10 m, and spatially aligned to a common grid. Figure 1 shows the spatial distribution of the four selected landslide study areas.

To further characterize the sample composition of the selected regions, both patch-level and pixel-level class distributions were calculated for the four regions, as shown in Table 1.

As shown in Table 1, a total of 6290 samples were included in the experiments, comprising 3151 landslide samples and 3139 non-landslide samples. The proportion of landslide samples in the four regions ranges from 49.3% to 54.8%, indicating a generally balanced patch-level class composition. At the pixel level, however, landslide pixels occupy a relatively small proportion of the image patches, ranging from approximately 4.2% in Italy to 7.5% in Itogon, whereas non-landslide pixels account for 92.5–95.8%. Overall, the four regions exhibit similar patch-level class proportions but different pixel-level landslide coverage, providing a more detailed characterization of the sample composition used for pixel-wise landslide segmentation.

The 6290 samples were randomly divided into training and validation sets at a ratio of 7:3, with 70% allocated to model training and the remaining 30% to model validation. The training–validation split was fixed throughout all experiments and remained unchanged across different comparative models and random-seed runs. The training set was used for model parameter optimization, while the validation set was used to monitor and evaluate model performance without participating in gradient-based parameter updating. The validation set was not used for hyperparameter tuning or epoch selection.

### 2.2. Landslide Detection Model: ConFormer-Net

**Figure 2:**
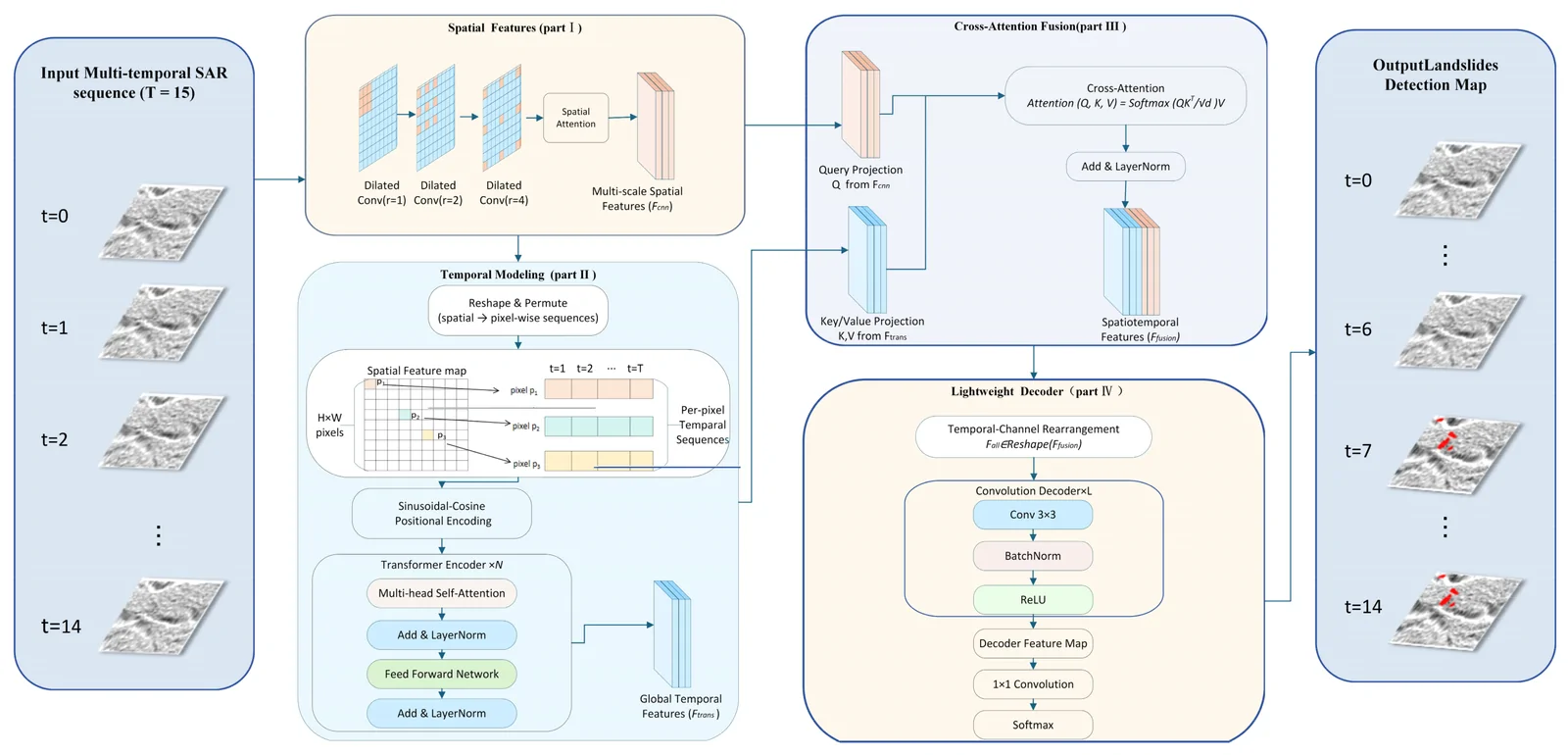
Architecture of the ConFormer-Net model for landslide detection. The model takes a sequence of multi-temporal SAR images comprising 15 time steps as input. First, a dilated convolutional encoder extracts local multiscale spatial features, while temporal positional encoding incorporates chronological information into the feature representations. Next, the Transformer encoder models global temporal dependencies, and the cross-attention fusion module adaptively integrates the spatial and temporal features. Finally, the decoder refines the fused spatiotemporal features to generate the landslide detection results.

To address the characteristics of multi-temporal SAR-based landslide detection, including substantial variations in landslide patch size, irregular boundary morphology, and changes in backscatter intensity before and after landslide occurrence, this study proposes the ConFormer-Net landslide detection model. The model is designed according to the workflow of “spatial feature extraction–temporal feature modeling–adaptive spatiotemporal feature fusion–pixel-level decoding.” First, the input multi-temporal SAR imagery is processed by a multiscale spatial feature extraction module to extract local morphological features of landslides. This module employs cascaded dilated convolutions with dilation rates of r = 1, 2, and 4 to enlarge the receptive field while preserving spatial resolution, thereby capturing spatial structures at different scales, such as landslide rear scarps, lateral boundaries, and depositional boundaries. A spatial attention mechanism is further introduced to adaptively weight discriminative spatial regions while suppressing noise interference caused by mountain shadows, bare ground, and complex terrain backgrounds. The overall architecture of ConFormer-Net is shown in Figure 2.

#### 2.2.1. Multiscale Spatial Feature Extraction Module Based on Dilated Convolution

**Figure 3:**
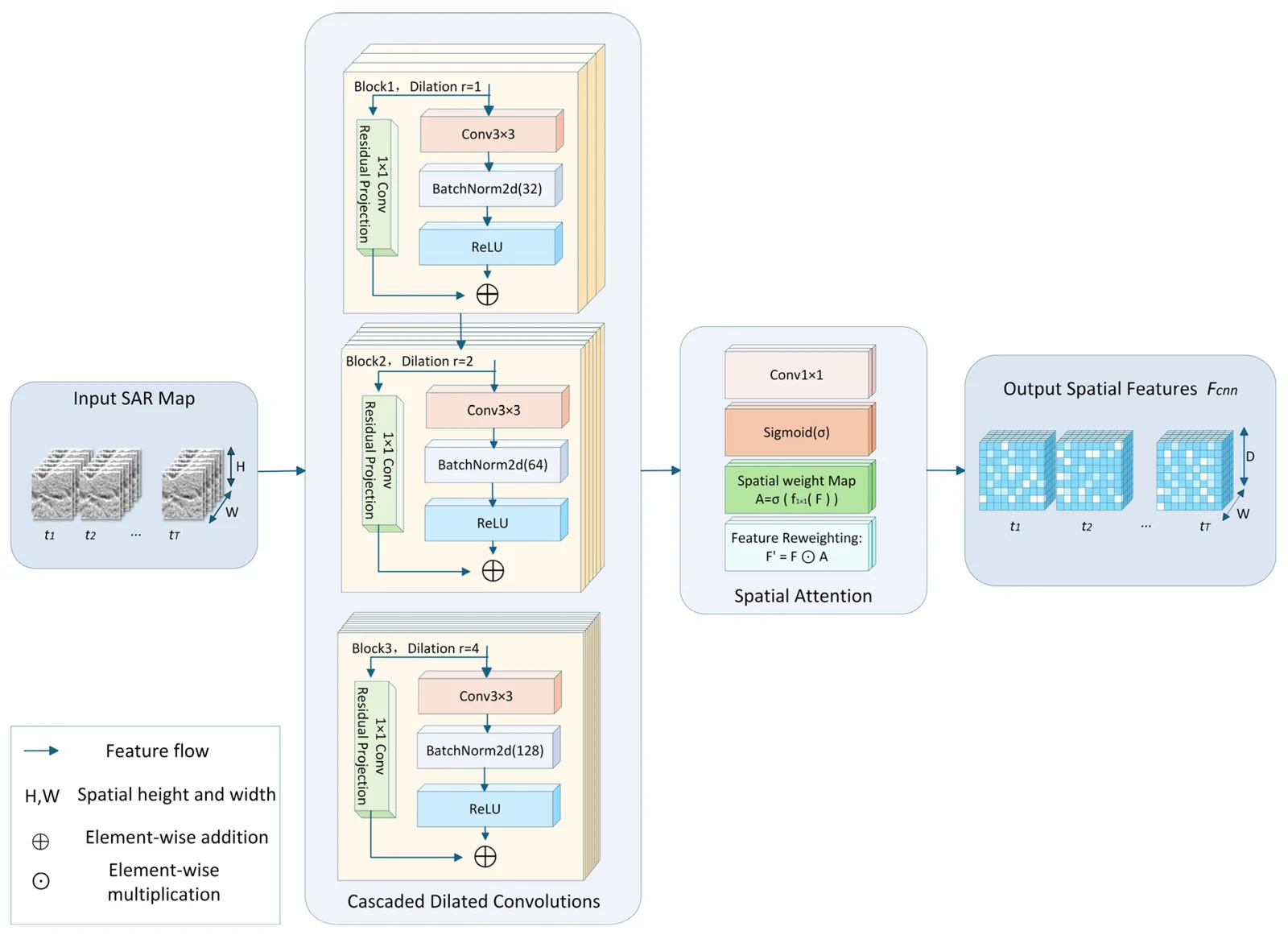
Flowchart of the dilated convolution-based multiscale spatial feature extraction module. The module first employs dilated convolutional layers with dilation rates of r = 1, 2, and 4 to construct multiscale receptive fields, enabling the extraction of landslide features at different scales without reducing spatial resolution. The multiscale features are then adaptively enhanced through a spatial attention mechanism to obtain the spatial feature representation *F_cnn_*.

Landslide rear scarps, lateral cracks, and depositional boundaries often exhibit banded, arcuate, or irregular patch-like spatial patterns. Conventional 3 × 3 convolutions primarily focus on local neighborhood textures and therefore struggle to simultaneously capture fine edge cracks and the overall extent of landslide areas, which may lead to incomplete representation of landslide boundaries. To address this limitation, dilated convolutions, as illustrated in Figure 3, are introduced to construct a multiscale feature extraction mechanism. By enlarging the receptive field without reducing spatial resolution, this mechanism enables the model to capture detailed landslide boundary information more effectively. In addition, vegetation, terrain shadows, road cut slopes, and speckle noise in SAR imagery may produce spatial features similar to those of landslides. Therefore, a spatial attention mechanism is further incorporated to adaptively reweight feature responses, thereby enhancing the spatially discriminative features of landslide regions.

**Figure 4:**
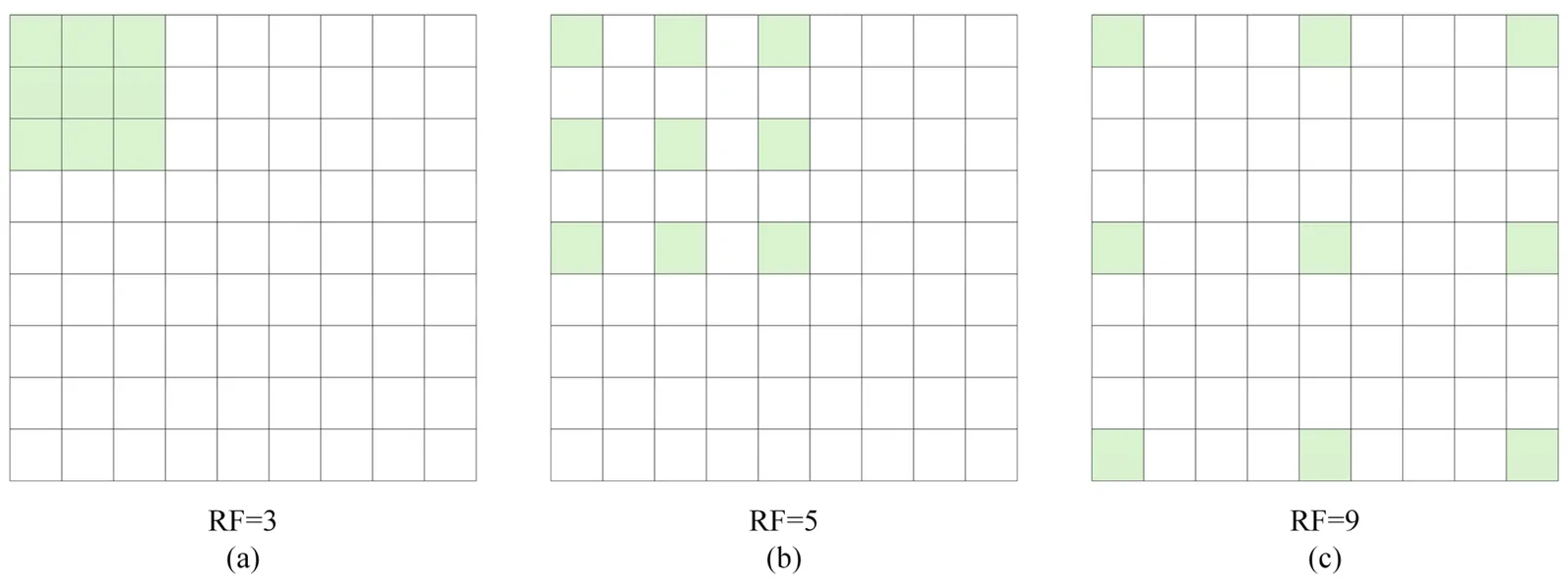
Comparison of the receptive fields of standard convolution and dilated convolution: (**a**) standard convolution; (**b**) dilated convolution with a dilation rate of 2; (**c**) dilated convolution with a dilation rate of 4.

In the Cascaded Dilated Convolutions module, 3 × 3 dilated convolutions [44] are combined with residual connections. The three convolutional blocks output 32, 64, and 128 feature channels, respectively. Each convolutional block consists of a dilated convolution, batch normalization, a ReLU activation function, and a 1 × 1 projection-based residual connection. This design allows the receptive field to be flexibly enlarged without reducing the spatial resolution. The receptive field (RF) of a single dilated convolutional layer is calculated using Equation (1), while the receptive fields corresponding to the three dilation rates are illustrated in Figure 4. Specifically, a standard convolution is first employed to aggregate information from neighboring pixels, after which the dilation rate is progressively increased to mitigate information loss during dilated convolution. Ultimately, the theoretical receptive field is expanded to a 15 × 15-pixel region, enabling the model to capture detailed features such as landslide boundaries.

(1) $$\begin{array}{c} RF=k+(k-1)(r-1) \end{array}$$

where *k* denotes the kernel size, and *r* denotes the dilation rate, defined as the pixel spacing between adjacent sampling points in the dilated convolution.

Furthermore, to reduce interference from complex background scattering associated with vegetation cover, terrain shadows, roads, and bare ground, a spatial attention mechanism is introduced after the cascaded dilated convolution module. The output features of the cascaded dilated convolution module are passed through a 1 × 1 convolution and a Sigmoid function to generate a spatial attention weight map. Element-wise multiplication is then applied to adaptively enhance feature responses in landslide regions while suppressing those in irrelevant background areas. The calculation process is expressed as follows:

(2) $$\begin{array}{c} A\;=\;\sigma \;{(\;f}_{1\times 1}(\;F\;)\;) \end{array}$$

(3) $$\begin{array}{c} F^{\prime }=F\;⨀\;A \end{array}$$

where *F* denotes the spatial feature map output by the cascaded dilated convolution module, *A* denotes the spatial attention weight map, f_1×1_(·) denotes the 1 × 1 convolutional mapping, *σ* denotes the Sigmoid activation function, and ⊙ denotes element-wise multiplication.

#### 2.2.2. Pixel-Level Temporal Modeling and Positional Encoding Mechanism

**Figure 5:**
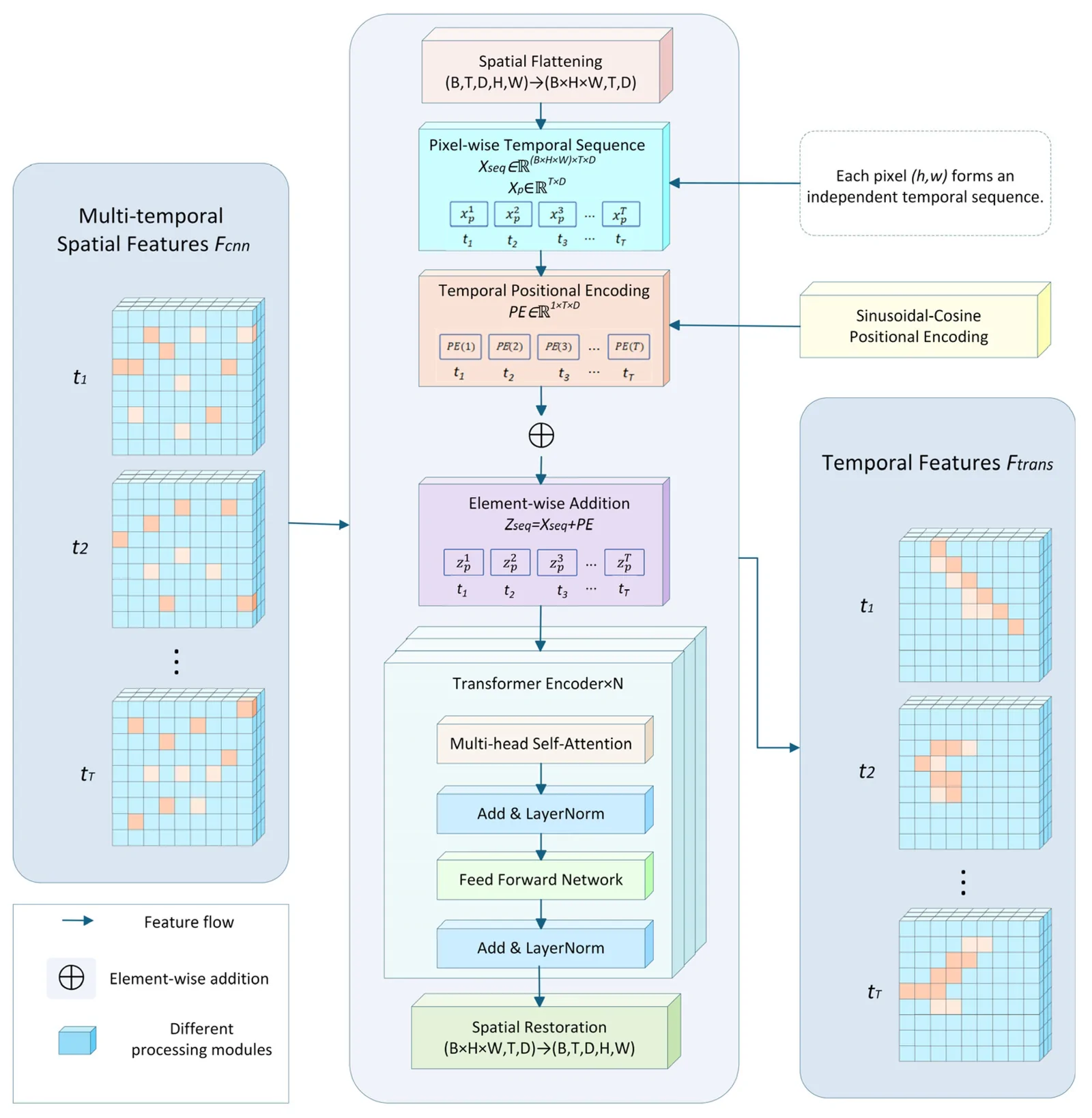
Architecture of the Transformer-based temporal modeling module. The multi-temporal spatial feature Fcnn is first flattened into pixel-level time series, so that each pixel location forms an independent temporal sample. Sinusoidal positional encoding is then added to preserve temporal order information, and the encoded sequence is fed into the Transformer encoder to model long-range dependencies across time steps, ultimately generating the temporal feature *F_trans_*.

Landslide detection depends not only on spatial morphological features, but also on the ability to model the temporal patterns of pre-event stable conditions, post-event disturbance responses, and their continuous evolution in multi-temporal SAR observations. Through the self-attention mechanism, the Transformer directly establishes relationships among features at different time steps and captures long-range temporal dependencies through parallel computation. However, the self-attention mechanism itself lacks an inherent awareness of temporal order. Therefore, this study introduces the sinusoidal positional encoding shown in Equation (4), which embeds temporal position information into the pixel-level temporal feature sequences. This enables the model to distinguish temporal feature differences among pre-event, post-event, and intermediate observation stages while modeling long-range temporal dependencies, thereby improving its ability to capture the temporal evolution of landslides [45]. The workflow of the Transformer-based temporal feature modeling module is illustrated in Figure 5.

(4) $$\begin{array}{c} PE\;(pos,2i)=sin\;(\frac{\mathrm{pos}}{{10,000}^{2i/D}}),\;PE\;(pos,2i+1)=cos\;(\frac{\mathrm{pos}}{{10,000}^{2i/D}}) \end{array}$$

where *pos* denotes the position of the time step, *i* represents the index of the feature dimension, and *D* denotes the feature dimension.

The Transformer encoder consists of one encoder layer with four attention heads, an embedding dimension of 128, and a feed-forward dimension of 64. A dropout rate of 0.1 and LayerNorm are used in the Transformer encoder. In the temporal positional encoding module, the multi-temporal spatial features output by the spatial feature extraction module are first unfolded according to their spatial locations into pixel-level temporal sequences *X_seq_ ∈ ℝ^(B×H×W)×T×D^*, such that the feature vectors of each pixel across different SAR observations form an independent temporal sequence. The sinusoidal positional encoding *PE ∈ ℝ^1×T×D^* is then embedded into the pixel-level temporal features through element-wise addition, yielding *Z_seq_ = X_seq_ + PE*. Through this operation, the features at each SAR time step retain not only the local spatial information extracted by the convolutional module, but also the corresponding temporal positional information. This enables the Transformer to distinguish different observation time steps during multi-head self-attention computation and to establish temporal associations between pre-event and post-event features. Finally, the features modeled by the Transformer Encoder are reshaped to restore the original spatial structure of the imagery, producing the temporal feature representation F_trans_.

#### 2.2.3. Spatiotemporal Feature Fusion Mechanism Based on Cross-Attention

A standalone CNN or Transformer architecture cannot simultaneously achieve detailed local spatial representation and long-range temporal dependency modeling for multi-temporal landslide detection. Conventional feature fusion methods typically rely on stacking, concatenation, or weighted summation. These methods lack adaptive capability and cannot effectively model the relative importance of different features, potentially resulting in information redundancy or the loss of critical features [46]. Therefore, this study introduces a cross-attention mechanism to enable interactive feature fusion through attention weights, thereby improving the effectiveness of spatiotemporal feature fusion.

Let the spatial features extracted by the convolutional encoder be denoted by

(5) $$\begin{array}{c} F_{cnn}\in R^{B\times T\times D\times H\times W} \end{array}$$

The temporal features output by the Transformer encoder are denoted by

(6) $$\begin{array}{c} F_{trans}\in R^{B\times T\times D\times H\times W} \end{array}$$

where *B* denotes the batch size, *T* denotes the number of time steps, *D* denotes the feature dimension, and *H* and *W* denote the spatial height and width, respectively.

The spatial features extracted by the convolutional encoder are used as the Query, while the temporal features modeled by the Transformer are used as the Key and Value. To compute cross-attention over the temporal sequence corresponding to each pixel location, the spatial dimensions H × W are first flattened and merged into the batch dimension, yielding the Query, Key, and Value tensors:

(7) $$\begin{array}{c} Q=\mathrm{Reshape}(F_{\mathrm{cnn}})\in R^{(B\cdot H\cdot W)\times T\times D} \end{array}$$

(8) $$\begin{array}{c} K=\mathrm{Reshape}(F_{trans})\in R^{(B\cdot H\cdot W)\times T\times D} \end{array}$$

(9) $$\begin{array}{c} V=\mathrm{Reshape}(F_{\mathrm{trans}})\in R^{(B\cdot H\cdot W)\times T\times D} \end{array}$$

The similarity between the Query and Key is computed to establish associations between spatial structural information and temporal variation information. The resulting attention weights are then used to achieve adaptive spatiotemporal feature fusion:

(10) $$\begin{array}{c} Attention\;(Q,\;K,\;V)=Softmax\;(\frac{QK^{T}}{\sqrt{d_{k}}})\;V \end{array}$$

where *d_k_* denotes the feature dimension of the Key vectors. The attention weights adaptively quantify the degree of association between the spatial features and the temporal features at different time steps, thereby enhancing the model’s ability to represent landslide evolution patterns. LayerNorm is applied to the output of the cross-attention module to stabilize feature representations during training.

Finally, the fused features are reshaped to restore the original spatial structure, yielding

(11) $$\begin{array}{c} F_{fusion}\in R^{B\times T\times D\times H\times W}\; \end{array}$$

Therefore, this module does not simply concatenate or add the CNN and Transformer features. Instead, it establishes a guidance mechanism in which spatial features guide the selection and integration of temporal features at the pixel-level temporal sequence. The CNN features provide local spatial structural cues, such as landslide rear scarps, lateral margins, and depositional boundaries, whereas the Transformer features characterize temporal dependencies between pre-event stable conditions and post-event disturbance responses. Through cross-attention computation, the model adaptively integrates local spatial morphological information with multi-temporal variation information, thereby improving its ability to distinguish landslide regions from non-landslide backgrounds in complex mountainous environments.

#### 2.2.4. Lightweight Convolutional Decoder for Pixel-Wise Semantic Segmentation

After spatial feature extraction, temporal modeling, and spatiotemporal feature fusion, the model must transform the fused high-dimensional spatiotemporal features into pixel-wise segmentation results. Therefore, this study designs a lightweight convolutional decoder to map the fused features to pixel-level classification results.

Since two-dimensional convolutional decoders generally process four-dimensional feature tensors, the temporal and feature dimensions are merged and reorganized. Specifically, the fused features corresponding to different time steps are unfolded along the channel dimension to obtain an all-temporal spatiotemporal fused feature representation:

(12) $$\begin{array}{c} F_{all}=\mathrm{Reshape}(F_{fusion})\in R^{B\times (T\cdot D)\times H\times W} \end{array}$$

This operation jointly encodes the fused features from all time steps along the channel dimension, ensuring that each spatial location contains the complete spatiotemporal fusion information of the entire temporal sequence. Consequently, the subsequent decoder can simultaneously utilize spatial structural information, temporal variation information, and their interactions across multiple time steps during pixel-level prediction. On this basis, F_all_ is fed into the lightweight convolutional decoder, where a 3 × 3 convolutional layer with 32 output channels, followed by BatchNorm and ReLU, further integrates the spatiotemporal information along the channel dimension to obtain the decoded features:

(13) $$\begin{array}{c} F_{\mathrm{dec}}=\mathrm{Decoder}(F_{all}) \end{array}$$

Then, a 1 × 1 convolutional layer is used to map the decoded features into the category space, producing the pixel-wise segmentation output:

(14) $$\begin{array}{c} Y={Conv}_{1\times 1}(F_{dec})\;\in R^{B\times K\times H\times W} \end{array}$$

where *K* denotes the number of classes, corresponding to landslide and non-landslide classes in the pixel-wise semantic segmentation task. Finally, the Softmax function is applied to convert the classification output into a pixel-wise class probability distribution:

(15) $$\begin{array}{c} P=\mathrm{Softmax}(Y) \end{array}$$

By incorporating the temporal dimension into the channel dimension and performing unified convolutional decoding, this decoding strategy preserves complete spatial features and temporal information, which helps improve the stability and robustness of landslide detection in complex terrain environments.

## 3. Results and Analysis

### 3.1. Experimental Setting and Evaluation Metrics

To ensure the reliability of the detection accuracy of the final model, all experiments in this paper were conducted under the same hardware and software conditions. The detailed configuration of the experimental environment is presented in Table 2.

Meanwhile, this paper adopted PyTorch with Python 3.8 as the deep learning framework. For all comparative experiments, the same data split, input data, preprocessing procedure, and evaluation metrics were adopted to ensure consistency in the experimental settings. Specifically, the number of training epochs was set to 200, the batch size was set to 4, and all input images were uniformly resized to a resolution of 128 × 128 pixels. The initial learning rate was set to 0.001, and a weight decay of 0.0005 was applied to prevent overfitting. The Adam optimizer and cross-entropy loss were used for model optimization. The learning rate remained fixed throughout training, and no additional data augmentation was applied. Model parameters were initialized using the default PyTorch initialization.

To evaluate the performance of the proposed ConFormer-Net model in landslide detection, accuracy, precision, recall, and F1-score were adopted to assess the detection accuracy of the model. The corresponding formulas are as follows:

(16) $$\begin{array}{c} \mathrm{Accuracy}=\frac{TP+TN}{TP+TN+FP+FN} \end{array}$$

(17) $$\begin{array}{c} \mathrm{Precision}=\frac{TP}{TP+FP} \end{array}$$

(18) $$\begin{array}{c} \mathrm{Recall}=\frac{TP}{TP+FN} \end{array}$$

(19) $$\begin{array}{c} F1=2\times \frac{Precision\times Recall}{Precision+Recall} \end{array}$$

In these formulas, TP, TN, FP, and FN denote the numbers of pixel-level true positives, true negatives, false positives, and false negatives, respectively. Accuracy represents the proportion of correctly predicted samples among all samples. Precision represents the proportion of true positive samples among the samples predicted as positive. Recall represents the proportion of actual positive samples that are correctly detected by the model. The F1-score jointly considers precision and recall and serves as a comprehensive metric for evaluating the model’s ability to accurately and completely identify positive samples. A lower F1-score generally indicates insufficient precision or recall, which may result from a low true positive detection rate or a high false positive detection rate. In contrast, a higher F1-score indicates that both precision and recall perform well, reflecting a more balanced classification result.

### 3.2. Visualization of Multi-Temporal SAR Samples

**Figure 6:**
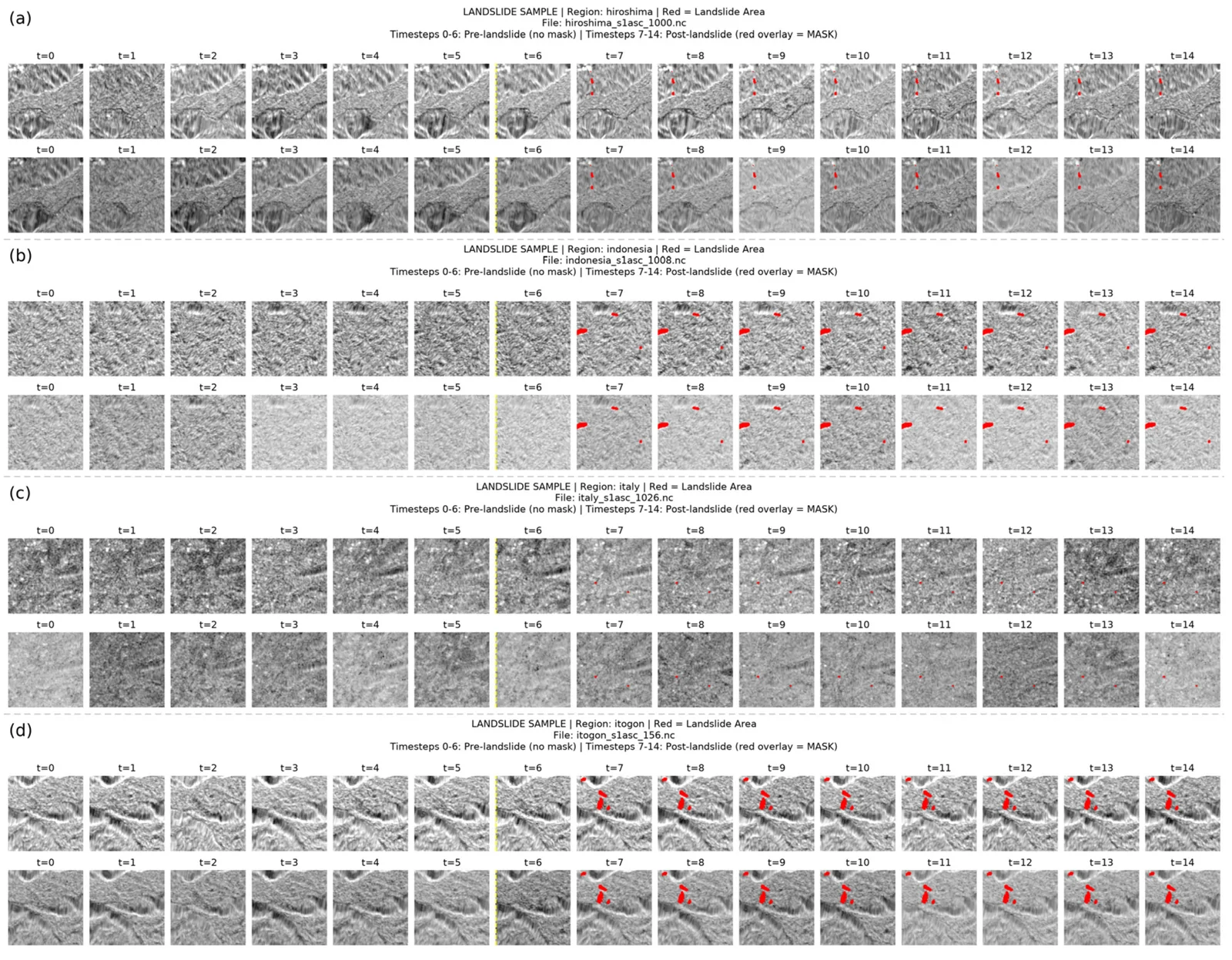
Visualization of representative multi-temporal SAR samples from different regions. (**a**) Hiroshima; (**b**) Indonesia; (**c**) Italy; and (**d**) Itogon. In the figure, the upper row presents VV-polarized imagery, while the lower row presents VH-polarized imagery. Each sample group contains complete temporal observations from t = 0 to t = 14. Specifically, t = 0 to t = 6 correspond to the pre-landslide image sequence without landslide masks, whereas t = 7 to t = 14 correspond to the post-landslide image sequence. The red regions indicate the ground-truth landslide masks.

Figure 6 presents representative multi-temporal SAR landslide samples selected from Hiroshima, Indonesia, Italy, and Itogon in the Sen12Landslides dataset. Each sample contains a complete sequence of 15 temporal observations, which is used as the input to ConFormer-Net. The first seven observations (t = 0 to t = 6) correspond to the pre-landslide period, while the subsequent observations (t = 7 to t = 14) correspond to the post-landslide period. By using the complete multi-temporal sequence as input, the model can jointly extract spatial features and model temporal dependencies among SAR observations acquired at different time steps.

Figure 6a–d presents representative multi-temporal SAR landslide samples from four regions: Hiroshima, Indonesia, Italy, and Itogon. Each sample contains a time series of 15 observations. The images from t = 0 to t = 6 represent the pre-landslide period with no landslide activity, and the images from t = 7 to t = 14 represent the post-landslide period. The red binary masks indicate the ground-truth landslide extents. From the perspective of SAR imaging mechanisms, landslide occurrence disrupts the original vegetation cover and slope structure. Exposed soil, accumulated rock debris, and changes in slope-surface roughness alter the surface backscattering characteristics, causing landslide areas in post-event images to exhibit backscatter responses distinct from the stable pre-event background. The dilated convolution module enlarges the receptive field to extract multiscale spatial contextual information, facilitating the characterization of landslide morphology and its differences from the surrounding background. Meanwhile, the Transformer-based temporal modeling module captures long-range temporal dependencies among SAR observations acquired at different time steps.

Unlike single-temporal SAR images, which are susceptible to interference from speckle noise and local topographic shadows, multi-temporal SAR image sequences provide continuous observations of backscatter changes before and after landslide occurrence. This enables the model to determine whether an observed anomaly exhibits temporal persistence and stage-specific characteristics, rather than classifying it solely on the basis of local brightness differences at a single time step. Consequently, ConFormer-Net can consistently identify the non-landslide state before landslide occurrence and maintain spatially coherent detection of the affected area after the event, thereby improving landslide detection accuracy.

**Figure 7:**
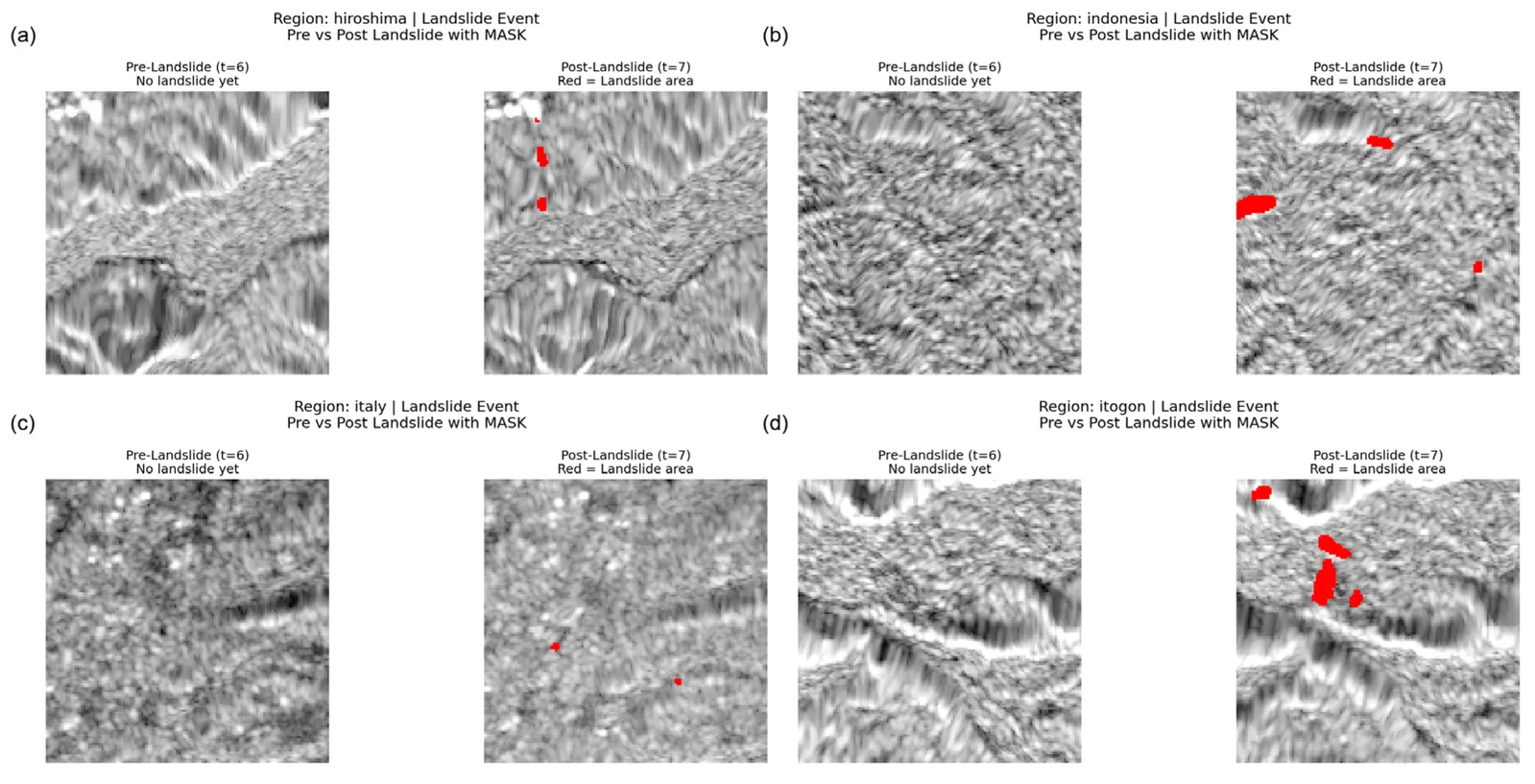
Visualization of representative pre- and post-landslide SAR samples from different regions. (**a**) Hiroshima; (**b**) Indonesia; (**c**) Italy; and (**d**) Itogon. In each group of images, the left panel shows the original terrain before landslide occurrence at *t* = 6, where no landslide activity is observed, while the right panel shows the post-landslide image at *t* = 7. The red regions denote the ground-truth landslide areas.

Figure 7 further presents representative SAR samples at two key time steps immediately before and after landslide occurrence, providing a direct comparison between the pre-landslide observation at t = 6 and the post-landslide observation at t = 7. This visualization complements the complete temporal sequences shown in Figure 6 and provides a more focused view of the SAR observations around the landslide event.

To further evaluate the ability of ConFormer-Net to distinguish non-landslide areas, temporal image sequences without landslide occurrence were selected as negative samples from the four study regions used above, namely Hiroshima, Indonesia, Italy, and Itogon. Unlike the landslide samples, these areas exhibit no evident landslide-induced disturbances throughout the complete temporal sequence and can therefore be used to assess false detections under complex terrain, heterogeneous background textures, and SAR speckle noise.

**Figure 8:**
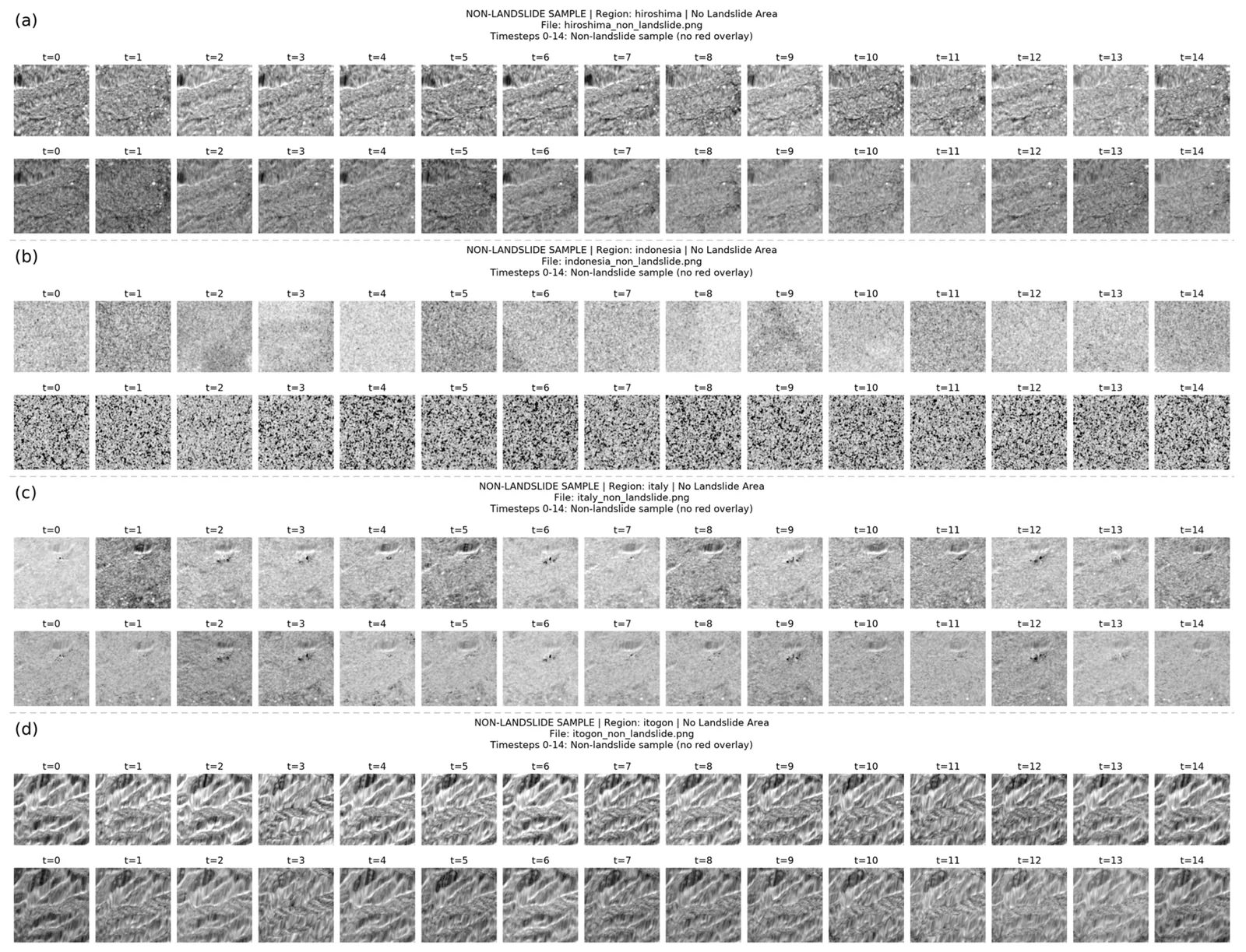
Visualization of representative multi-temporal SAR non-landslide samples from different regions (**a**) Hiroshima; (**b**) Indonesia; (**c**) Italy; and (**d**) Itogon. No landslides occurred in the sample areas during the observation periods.

As shown in Figure 8, no landslide areas are detected in the four representative non-landslide samples from Hiroshima, Indonesia, Italy, and Itogon. The temporal sequences of these samples remain relatively stable throughout the observation period without evident landslide-induced disturbances. By jointly modeling multiscale spatial features and temporal dependencies, ConFormer-Net can distinguish these non-landslide samples from landslide-affected areas under complex background conditions. The false-positive performance of the model is further quantitatively evaluated using the FPR in the leave-one-region-out experiment presented in Section 3.4.

In addition, to evaluate the detection performance for landslides of different sizes, landslide samples were divided into three categories according to their area: small (<1000 m^2^), medium (1000–5000 m^2^), and large (>5000 m^2^). The F1-score was calculated separately for each size category. Boundary F1-score was further used to evaluate the accuracy of landslide boundary delineation. The samples were stratified according to landslide area, and the F1-score and Boundary F1-score were calculated for each size category, as shown in Table 3.

As shown in Table 3, the F1-scores for small, medium, and large landslides were 91.20%, 95.50%, and 96.80%, respectively. Although the detection performance decreased with decreasing landslide size, the model still achieved an F1-score above 90% for small landslides. The corresponding Boundary F1-scores were 82.50%, 87.30%, and 90.10%, respectively, showing a similar increasing trend with landslide size. These results indicate that the model maintains relatively stable detection performance across different landslide sizes, while accurate boundary delineation remains more challenging for small landslides.

**Figure 9:**
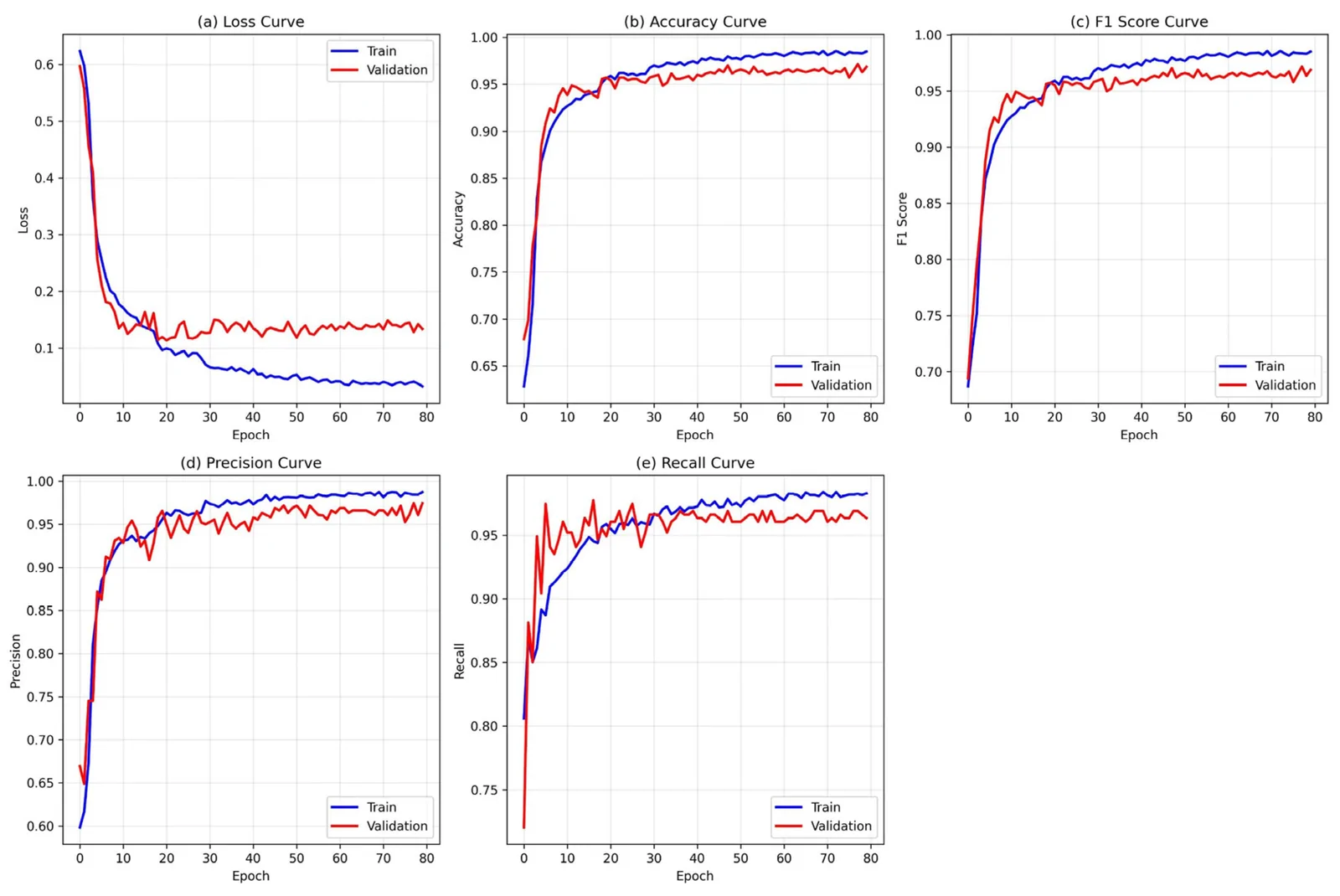
Training and validation loss curves and evaluation metrics of the ConFormer-Net model. (**a**) Loss curves of the training and validation sets; (**b**) accuracy curves of the training and validation sets; (**c**) F1-score curves of the training and validation sets; (**d**) precision curves of the training and validation sets; and (**e**) recall curves of the training and validation sets.

To evaluate the stability and convergence of the model, the training and validation loss curves in Figure 9a, accuracy curves in Figure 9b, F1-score curves in Figure 9c, precision curves in Figure 9d, and recall curves in Figure 9e were analyzed. Both the training and validation losses show an overall decreasing trend and gradually stabilize after approximately 80 epochs, indicating that the model can rapidly learn effective discriminative features from multi-temporal SAR imagery without exhibiting evident overfitting. The accuracy, F1-score, precision, and recall curves remain at relatively high levels during the later stages of training, demonstrating that the model can accurately detect landslide regions while effectively reducing false detections in non-landslide areas. These results indicate that ConFormer-Net achieves a favorable balance between high precision and high recall in landslide detection, ensuring accurate delineation of landslide regions while reducing missed detections, thereby further validating the effectiveness of the proposed model.

### 3.3. Comparative Experiments

To verify the effectiveness of ConFormer-Net in landslide detection, CNN, CNN-LSTM, GRU, ConvLSTM, and CNN3D were selected as comparative models. In addition, a ResNet50 baseline with approximately 850 K parameters, comparable to the 802 K parameters of ConFormer-Net, was introduced to further examine whether the performance advantage of ConFormer-Net remained after controlling for model size. CNN, as a representative spatial feature extraction model, primarily relies on local spatial texture and morphological information to detect landslide regions. CNN-LSTM and GRU are sequential temporal modeling approaches that typically extract spatial features first and then model temporal information through recurrent structures. ConvLSTM and CNN3D are spatiotemporal joint modeling methods based on local convolutional operations, enabling them to capture spatial and temporal variations within local neighborhoods. The F1-score, accuracy, precision, and recall of each model were evaluated over five independent runs using different random seeds (42, 123, 2024, 888, and 10,086), while keeping the training and validation sets fixed. The results are expressed as the mean ± standard deviation, and the number of model parameters was recorded to evaluate model complexity. Statistical significance was further assessed using a two-sided paired *t*-test based on the F1-scores obtained from the five runs, with each comparative model tested against ConFormer-Net. A *p*-value of less than 0.05 was considered statistically significant. The comparative experimental results are presented in Table 4.

As shown in Table 4, ConFormer-Net achieved the best overall performance among all comparative models, with an average F1-score of 95.35, accuracy of 95.37, precision of 96.85, and recall of 93.85. The relatively small standard deviations indicate that ConFormer-Net maintained stable performance across different random-seed runs. The statistical significance analysis further showed that the differences in F1-score between ConFormer-Net and the comparative models were statistically significant (*p* < 0.05). Compared with CNN, which relies solely on spatial information modeling, ConFormer-Net improved the average F1-score by 10.14 percentage points. Although CNN achieves the highest recall of 97.55%, its precision is only 75.65%, indicating that the model can identify most true landslide areas but also produces a considerable number of false detections. Bare ground, road-cut slopes, and other exposed surfaces in complex mountainous environments may exhibit local texture and spectral or scattering characteristics similar to those of landslides, thereby increasing the risk of false detections by deep learning models. For SAR imagery, terrain shadows, layover, and speckle noise may further increase the confusion between landslide and non-landslide backgrounds. Because CNN primarily relies on intensity, texture, and spatial structural information within limited local neighborhoods for discrimination, non-landslide areas exhibiting local backscatter anomalies similar to those of landslides can be incorrectly identified as landslides. In addition, a standalone CNN lacks temporal modeling of the pre-event stable state and persistent post-event changes, making it difficult to determine whether a local anomaly represents a persistent surface change caused by a landslide or a transient response induced by short-term environmental variations, topographic effects, or SAR noise. Therefore, although CNN achieves high recall with relatively few missed detections, it also incorrectly identifies more non-landslide areas with similar local characteristics as landslides, resulting in an increased number of false positives and reduced precision.

Compared with the sequential temporal models CNN-LSTM and GRU, ConFormer-Net improves the F1-score by 1.67 and 1.90 percentage points, respectively. These results indicate that, although such models incorporate temporal modeling, they generally first use CNNs to extract spatial features from images at different time steps and then model temporal dependencies through recurrent structures. Because temporal information is propagated progressively between adjacent time steps through hidden states, information attenuation may occur in long sequences. Consequently, these models may have difficulty fully capturing long-range temporal dependencies for global temporal modeling and adaptively integrating local spatial features with global temporal variation information.

Compared with local convolution-based spatiotemporal models, including ConvLSTM and CNN3D, ConFormer-Net improves the F1-score by 3.05 percentage points. This indicates that, although ConvLSTM and CNN3D can jointly model spatial and temporal information to some extent, their spatiotemporal feature extraction still relies primarily on local convolutional windows. Their receptive fields are therefore limited, and the interaction between spatial and temporal information is largely confined to local neighborhoods, making it difficult to effectively model global temporal dependencies. Notably, ConvLSTM achieves a precision of 97.35%, slightly higher than that of ConFormer-Net, but its recall is only 87.70%, indicating a greater number of missed detections. In contrast, ConFormer-Net increases recall by 6.15 percentage points while maintaining high precision, thereby achieving a better balance between precision and recall and ultimately obtaining a higher F1-score.

Importantly, the parameter-matched residual baseline contains approximately 850 K parameters, which is higher than the 802 K parameters of ConFormer-Net. Nevertheless, ConFormer-Net achieves an F1-score of 95.35%, exceeding that of the parameter-matched residual baseline (87.50%) by 7.85 percentage points, while its accuracy is higher by 9.77 percentage points. Although the residual baseline achieves a higher recall, its substantially lower precision results in a markedly lower F1-score. These results demonstrate that the performance advantage of ConFormer-Net persists when model size is controlled, suggesting that the improvement cannot be explained solely by an increase in the number of model parameters. Instead, the joint modeling of multiscale spatial features, long-range temporal dependencies, and their adaptive fusion contributes substantially to the improved overall detection performance.

To address the limitations of the comparative models, ConFormer-Net employs dilated convolutions to enlarge the receptive field and enhance multiscale spatial feature representation. A Transformer encoder is used to model multi-temporal SAR sequences and capture global dependencies across long temporal ranges. Furthermore, a cross-attention mechanism is introduced to jointly model and fuse spatial and temporal features, enabling the adaptive integration of local spatial information with global temporal variation information. These designs demonstrate the advantages of ConFormer-Net in multiscale spatial feature extraction, long-range temporal dependency modeling, and spatiotemporal feature fusion.

To further evaluate the model complexity and computational efficiency of different models, the number of parameters, FLOPs, inference time, training time, and peak GPU memory consumption were compared in the same experimental environment. FLOPs and inference time were measured for a single input sample, with the inference time evaluated using a batch size of 1. Training time and peak GPU memory consumption were measured under the training configuration described in Section 3.1. The results are presented in Table 5.

As shown in Table 5, ConFormer-Net requires greater computational resources than the comparative models, with 7.63 G FLOPs, an inference time of 31.4 ms per sample, a training time of 7.8 h per run, and a peak GPU memory consumption of 5420 MB. Combined with the performance results in Table 4, ConFormer-Net achieves the best overall detection performance, but at the cost of higher computational overhead. Notably, ResNet50 contains more parameters than ConFormer-Net, yet its F1-score is 7.85 percentage points lower. These results indicate that the performance advantage of ConFormer-Net cannot be attributed solely to model size and demonstrate a clear trade-off between detection performance and computational efficiency.

Overall, the comparative experiments demonstrate the advantages of the proposed ConFormer-Net for landslide detection. The model not only outperforms the comparative methods across multiple evaluation metrics, but also achieves a more favorable balance between detection accuracy and stability.

### 3.4. Leave-One-Region-Out Experiment

To further examine the spatial independence and cross-region generalization capability of ConFormer-Net, a leave-one-region-out (LORO) experiment was conducted. In each experimental run, all samples from one of the four study regions were completely held out, while samples from the remaining three regions were used for model training. This procedure was repeated four times, with Hiroshima, Indonesia, Italy, and Itogon successively used as the held-out region. Because the training samples and the held-out samples originated from geographically distinct regions, spatially neighboring patches from the held-out region were completely excluded from model training. In addition to F1-score, detailed pixel-level metrics, including IoU, Dice, precision, recall, specificity, and false-positive rate (FPR), were calculated for each held-out region.

The results of the LORO experiment are presented in Table 6. ConFormer-Net achieved F1-scores of 90.10–94.68% across the four held-out regions, with an average F1-score of 92.54%. The average precision, recall, and IoU were 94.18%, 90.98%, and 86.18%, respectively. The specificity remained above 98.7% for all regions, while the FPR ranged from 0.58% to 1.28%. Compared with the F1-score of 95.35% obtained under the original experimental setting, the average F1-score decreased by 2.81 percentage points under the more stringent cross-region setting. These results indicate that ConFormer-Net maintains stable pixel-level detection performance when applied to geographically unseen regions.

### 3.5. Temporal Shift Experiment

To examine whether ConFormer-Net relies on the fixed timing of landslide occurrence in the standardized temporal sequences, temporal-shift experiments were conducted using the same trained model, with only the timing of the landslide event within the input sequence changed. In the original setting, the landslide event was located at t = 7, while in the two shifted settings, the event time was moved to t = 4 and t = 5, respectively.

As shown in Table 7, ConFormer-Net achieved an F1-score of 95.27% under the original setting. When the landslide event time was shifted from t = 7 to t = 4 and t = 5, the F1-scores were 94.85% and 94.91%, corresponding to decreases of only 0.42 and 0.36 percentage points, respectively. Precision and recall also remained close to those obtained under the original setting. The limited performance degradation under the two temporal-shift settings indicates that ConFormer-Net does not strongly rely on the fixed timing of landslide occurrence in the standardized sequence and can still effectively capture landslide-related temporal changes when the event occurs at different temporal positions within the input sequence.

### 3.6. First Post-Event Acquisition Experiment

To evaluate the rapid post-event detection capability of ConFormer-Net, the model was evaluated using only the first post-event SAR image at t = 7 as input and compared with the complete temporal sequence from t = 0 to t = 14. The temporal input length was therefore set to T = 1 for the single-image setting, while the remaining experimental settings were kept unchanged.

The experimental results are presented in Table 8. When only the first post-event image at t = 7 was used, ConFormer-Net achieved an F1-score of 86.25%, a precision of 88.10%, and a recall of 84.50%. In comparison, the full temporal sequence achieved an F1-score of 95.27%, a precision of 96.79%, and a recall of 93.79%. The F1-score decreased by 9.02 percentage points when only the first post-event image was used. These results indicate that ConFormer-Net can produce landslide detection results once the first post-event SAR image becomes available, whereas the full temporal sequence provides more comprehensive temporal change information and achieves substantially higher detection performance.

### 3.7. Ablation Experiment

To systematically evaluate the contributions of different modules and architectural configurations in ConFormer-Net, a series of ablation experiments were conducted. The experiments focus on the spatiotemporal fusion strategy, temporal modeling, multiscale dilated convolution, spatial attention, positional encoding, dilation-rate configuration, temporal-sequence length, and decoder structure. For the cross-attention module, concatenation, summation, and gated fusion were adopted as alternative fusion strategies. The Transformer-based temporal modeling module was also removed or replaced with a unidirectional LSTM. In addition, the spatial and temporal components, as well as the decoder structure, were independently modified to further examine their effects on model performance. The results are presented in Table 9.

As shown in Table 9, replacing the cross-attention mechanism with concatenation, summation, and gated fusion results in F1-scores of 95.12%, 95.20%, and 95.15%, respectively, corresponding to decreases of 0.15, 0.07, and 0.12 percentage points compared with the full model. Although the differences among these fusion strategies are relatively small, the full model achieves the highest F1-score, indicating that cross-attention provides a modest improvement in the interaction and integration of spatial and temporal features.

Removing the temporal modeling module resulted in the largest performance decrease among the ablation variants, with the F1-score decreasing from 95.27% to 86.89%. Replacing the Transformer-based temporal modeling module with a unidirectional LSTM reduced the F1-score to 90.83%, indicating that, compared with recurrent temporal modeling, the Transformer provides a more effective representation of temporal dependencies. Since removing the temporal modeling module also substantially reduces the model parameter count, additional temporal ablations with the model size maintained at 802 K were conducted to further examine the role of temporal information. Removing positional encoding reduced the F1-score to 94.78%, indicating that incorporating temporal positional information enables the model to distinguish the chronological order of different observations and contributes to detection performance. In addition, shortening the input temporal sequence from 15 time steps to 10 and 5 reduced the F1-score to 94.45% and 92.10%, respectively, indicating that temporal-sequence length affects model performance. These results further demonstrate that the performance contribution associated with temporal modeling cannot be explained solely by differences in model capacity. Overall, the Transformer-based temporal modeling strategy, positional encoding, and the length of the input temporal sequence all contribute positively to the performance of ConFormer-Net. The Transformer establishes dependencies among observations at different time steps through self-attention and incorporates positional encoding to represent their temporal order, thereby enhancing the modeling of long-range temporal dependencies and temporal variation patterns in multi-temporal SAR imagery.

The spatial-component ablations further demonstrate the contribution of multiscale spatial feature extraction. Replacing the cascaded dilated convolutions with standard convolution using only r = 1 decreases the F1-score by 1.07 percentage points, indicating that multiscale receptive fields contribute to the representation of landslide spatial features. Removing spatial attention results in a decrease of 0.42 percentage points. In addition, changing the original dilation-rate configuration of 1, 2, and 4 to 1, 2, and 8 or 1, 3, and 5 reduces the F1-score by 0.17 and 0.22 percentage points, respectively. These results suggest that the adopted dilation-rate configuration is more suitable among the evaluated settings.

The decoder ablation results show that increasing decoder complexity provides little additional benefit. Replacing the lightweight decoder with a heavier U-Net-like decoder increases the parameter count from 802 K to 1150 K but improves the F1-score by only 0.04 percentage points. In contrast, simplifying the decoder to a single-layer structure reduces the F1-score by 0.67 percentage points. These results indicate that the adopted lightweight decoder provides a favorable balance between model complexity and detection performance.

Overall, the expanded ablation experiments demonstrate that the performance of ConFormer-Net benefits from the complementary contributions of multiscale spatial feature extraction, temporal modeling, spatial attention, positional encoding, and spatiotemporal feature fusion. Temporal-related ablations produce the largest observed performance differences, while the parameter-count-preserving experiments further confirm that temporal order and sequence length contribute to model performance beyond differences in model capacity. Cross-attention provides a modest improvement over alternative fusion strategies, and the current dilation-rate and decoder configurations achieve favorable performance without introducing unnecessary model complexity.

## 4. Discussion

The significance of the experimental results lies not only in the superior overall performance achieved by ConFormer-Net, but also in its ability to overcome the limitations of CNNs in landslide detection. Although CNN achieves a recall of 97.74%, its precision is only 75.88%, indicating that it tends to overestimate landslide extents and achieves a high detection rate at the cost of numerous false positives. In contrast, ConFormer-Net increases precision to 96.79% while maintaining a recall of 93.79%, resulting in an F1-score of 95.27%. Figure 6, Figure 7 and Figure 8 present representative multi-temporal SAR samples processed by ConFormer-Net for landslide detection. By jointly extracting multiscale spatial features and modeling long-range temporal dependencies from the complete temporal sequences, the model identifies landslide and non-landslide states under different regional conditions. Together with the quantitative evaluation results, these representative samples illustrate the effectiveness of the proposed spatiotemporal modeling strategy for landslide detection.

### 4.1. Spatiotemporal Joint Modeling Enhances the Discrimination Between Landslide-Induced Changes and Background Disturbances

After a landslide occurs, the land cover and topographic structure of the affected area undergo substantial changes. The original vegetation is extensively damaged or removed, exposing soil, rock, and debris materials. In the landslide transition zone and coarse-grained debris accumulation zones, surface roughness generally increases due to slope failure, material transport, and subsequent redeposition, whereas accumulation zones dominated by fine-grained materials such as mud and silt tend to exhibit relatively smooth surfaces. Meanwhile, geomorphic units such as the rear scarp, landslide transition zone, and landslide deposits alter the local slope morphology and radar imaging geometry, potentially causing geometric distortions such as radar shadow and layover. Correspondingly, vegetation volume scattering weakens after landslide occurrence, while surface scattering from exposed soil, rock, and landslide deposits becomes more pronounced. Rough surfaces in the landslide transition zone and coarse-grained deposits can enhance diffuse scattering, whereas relatively smooth fine-grained deposits or water-covered surfaces are more likely to produce specular reflection. Steep terrain features, such as the rear scarp, may also cause locally enhanced backscatter, radar shadow, and double-bounce scattering. Consequently, Sentinel-1 backscatter after landslide occurrence exhibits pronounced spatial variability, with its response jointly controlled by vegetation cover, surface roughness, material properties, and local terrain geometry [47].

The physical changes induced by landslides are further reflected in variations in SAR backscatter intensity, spatial texture, and local geometric responses, which are transformed into temporal features that can be learned by the network. In ConFormer-Net, the multiscale convolution module first encodes the SAR images acquired at each observation time, representing differences in backscatter and spatial texture caused by vegetation destruction, exposed soil and rock, changes in surface roughness, and the formation of scarps and landslide deposits as spatial features at each time step. Subsequently, the features at the same spatial location across different observation times are organized into a pixel-level temporal sequence, enabling the scattering-state changes at that location before and after landslide occurrence to be compared along the temporal dimension. Before landslide occurrence, slope-surface cover and scattering conditions are relatively stable, and both adjacent and non-adjacent pre-event observations generally exhibit high feature consistency. After landslide occurrence, SAR responses caused by vegetation destruction, exposed soil and rock, changes in surface roughness, and alterations in local geometric structures cause the post-event features to deviate markedly from the pre-event state, with these differences remaining relatively persistent across multiple subsequent observations. Through self-attention, the Transformer computes the correlations among features from different observation times, allowing each time step to establish direct relationships with all other time steps in the entire temporal sequence [48]. In this way, the model can simultaneously characterize the relatively stable temporal relationships before landslide occurrence, the state transition associated with landslide occurrence, and the persistent evolution of post-event disturbance features.

ConFormer-Net employs cascaded dilated convolutions to enlarge the receptive field, allowing local disturbance textures, landslide boundaries, and more complete slope structures to be represented at multiple spatial scales, while spatial attention further enhances discriminative spatial features. In complex SAR scenes, terrain shadows, exposed surfaces, vegetation disturbances, and speckle noise may produce local backscatter or texture responses similar to those of landslide-affected areas, thereby increasing the difficulty of spatial discrimination. The Transformer-based temporal module further establishes dependencies among observations acquired at different time steps, enabling the model to incorporate multi-temporal variations before and after landslide occurrence into the discrimination process. By jointly modeling spatial structural features and temporal variation features, ConFormer-Net obtains a more comprehensive spatiotemporal representation, thereby improving the discrimination between landslide and non-landslide areas under complex background conditions.

CNN-LSTM, GRU, ConvLSTM, and CNN3D can all exploit temporal information to some extent and therefore perform substantially better than the purely spatial CNN, but they still underperform compared with ConFormer-Net. Recurrent models must progressively compress historical information into hidden states, which may weaken the stable background information from earlier time steps during long-sequence propagation. ConvLSTM and CNN3D, by contrast, focus more strongly on variations within local spatiotemporal neighborhoods and are less effective in modeling pre- and post-event relationships between temporally distant observations. Self-attention, however, can directly establish associations between any pair of time steps, enabling pre-event information and post-event responses to be compared without continuous recurrent propagation. Therefore, the experimental results reflect not merely an improvement in network-level accuracy, but a fundamental extension of the landslide detection criterion from isolated local texture responses to the joint discrimination of local spatial morphology and multi-temporal variation patterns.

### 4.2. The Critical Role of Temporal Dependency Modeling in Improving Model Performance

The ablation experiments further reveal the relative contributions of different modules to the performance improvement of the model. After removing the temporal modeling module, the F1-score decreases from 95.27% to 86.89%, representing a reduction of 8.38 percentage points. Accuracy and precision decrease by 9.86 and 11.79 percentage points, respectively, whereas recall increases from 93.79% to 95.48%. This asymmetric change is more informative than the decline in any single metric. Without temporal information, the model can still respond to numerous local anomalies, so the apparent detection rate does not decrease and even increases slightly. However, it can no longer determine whether these anomalies are actually caused by landslides, resulting in the extensive inclusion of non-landslide areas in the predicted masks. These results indicate that temporal modeling improves the model’s ability to discriminate between landslide and non-landslide regions by incorporating information from multiple observation times.

When the Transformer is replaced with a unidirectional LSTM, the F1-score decreases to 90.83%, indicating that “using temporal data” is not equivalent to “fully exploiting temporal relationships.” A unidirectional LSTM recursively updates its hidden state in chronological order, and subsequent predictions depend on historical information that has undergone repeated compression. Consequently, the direct correspondence between early pre-event conditions and later post-event responses may be weakened. In contrast, the Transformer enables global interactions among different time steps, while sinusoidal positional encoding further provides these interactions with explicit temporal order, allowing the model to distinguish stable, abrupt-change, and persistent-response stages. Therefore, positional encoding does more than simply label individual time steps; it enables the Transformer to perceive the chronological relationships among different observations during self-attention computation. For responses with similar local backscatter characteristics, their positions within the pre- and post-event sequences and their relationships with preceding and subsequent observations provide additional temporal information for feature discrimination.

In practical SAR observations, speckle noise, changes in soil moisture, seasonal vegetation fluctuations, and topographic effects may introduce short-term variations in backscatter characteristics. The temporal-sequence ablation experiments show that the length of the input temporal sequence affects detection performance, further supporting the importance of multi-temporal information for landslide detection.

### 4.3. The Incremental Optimization Effect of Cross-Attention on Spatiotemporal Feature Fusion

After removing the cross-attention mechanism, the F1-score decreases from 95.27% to 95.12%, while precision and recall decrease by 0.29 and 0.19 percentage points, respectively. Compared with the pronounced performance degradation caused by removing the temporal modeling module, eliminating cross-attention results in only minor declines across the evaluation metrics. This indicates that the module primarily provides incremental optimization of spatiotemporal feature fusion by adaptively measuring the associations between spatial morphological information and temporal variation features at different time steps. Although the numerical gain introduced by cross-attention is relatively limited, it still helps strengthen the consistency between spatial structural cues and temporal anomaly responses while reducing interference from irrelevant temporal variations. Therefore, the value of this module lies mainly in the coordinated organization and optimized fusion of the existing spatiotemporal information, rather than in independently identifying temporal changes associated with landslides.

In terms of its mechanism, cross-attention uses the spatial features extracted by the convolutional encoder as the Query and the temporal features encoded by the Transformer as the Key and Value. Attention weights are calculated from the similarity between the Query and Key and are subsequently used to perform weighted fusion of the temporal features. Compared with direct concatenation or fixed-weight fusion, this mechanism dynamically adjusts the contribution of temporal information from different time steps according to its association with the spatial features, thereby further optimizing the interaction between spatial structural information and multi-temporal variation information.

### 4.4. Limitations and Future Work

Despite the promising landslide detection performance achieved by ConFormer-Net, several limitations remain in practical applications. First, although the four study regions differ in terrain conditions, surface backgrounds, and landslide morphologies, their spatial coverage remains limited and cannot fully represent scenarios across different climatic zones, geological settings, and landslide triggering mechanisms. Second, the model is trained and validated using 128 × 128-pixel SAR image patches, whereas practical applications generally require detection over large-scale continuous imagery. Landslides spanning multiple patches or occurring near patch boundaries may be affected by image tiling, overlap settings, and result mosaicking, and the regional-scale detection stability therefore requires further validation.

In addition, layover, terrain shadow, speckle noise, seasonal vegetation changes, and human-induced disturbances such as mining, road-slope excavation, and terraced-land modification may increase confusion between landslide and non-landslide areas. Representative failure-case visualizations were not included in this study; future work will further incorporate related error analyses to better characterize the specific conditions under which false detections occur. Finally, because the temporal sequences are organized with reference to landslide event dates, the current model is primarily applicable to event-centered post-event landslide detection and cannot yet directly identify pre-failure deformation processes or predict landslide occurrence in advance. Continuous operational monitoring would therefore require additional temporal-window localization or change-point detection.

Future work will expand training samples across different climatic and geological settings and landslide types, improve regional-scale detection for continuous SAR imagery, and integrate optical imagery, LiDAR, InSAR deformation, rainfall, and other multi-source observations to enhance model adaptability and explore landslide deformation evolution and early warning.

## 5. Conclusions

To address the limitations of insufficient local spatial detail representation, restricted long-range temporal dependency modeling, and inadequate spatiotemporal feature fusion in landslide detection from multi-temporal SAR imagery, this study proposes ConFormer-Net, a landslide detection model based on the joint modeling of convolutional neural networks and Transformers. The model employs dilated convolutions to construct a multiscale spatial feature extraction module, thereby enlarging the receptive field while preserving spatial resolution. Sinusoidal positional encoding and a Transformer encoder are then used to model temporal order and long-range temporal dependencies in multi-temporal SAR imagery. Furthermore, a cross-attention mechanism adaptively fuses spatial and temporal features, thereby improving pixel-wise semantic segmentation of landslide areas under complex mountainous backgrounds.

Experimental results based on the Sen12Landslides dataset demonstrate that ConFormer-Net achieves strong overall performance, with an F1-score, accuracy, precision, and recall of 95.27%, 95.29%, 96.79%, and 93.79%, respectively. Comparative experiments and ablation experiments further verify the effectiveness of multiscale spatial feature extraction, Transformer-based temporal modeling, and cross-attention fusion in improving landslide detection performance. Overall, the proposed method provides an effective spatiotemporal joint-modeling framework for landslide detection from multi-temporal SAR imagery.

## Figures and Tables

**Table 1:** Patch-level and pixel-level class distributions in each study region.

Region	Total Number of Samples	Number of Landslide Samples	Number of Non-Landslide Samples	Proportion of Landslide Samples (%)	Landslide Pixels (%)	Non-Landslide Pixels (%)
Hiroshima	864	457	407	52.9	6.8	93.2
Indonesia	622	311	311	50.0	5.1	94.9
Italy	4576	2258	2318	49.3	4.2	95.8
Itogon	228	125	103	54.8	7.5	92.5
Total	6290	3151	3139	50.1	4.8	95.2

**Table 2:** Experimental Settings.

Devices	Configuration
Operating system	Ubuntu 22.04
CPU	Intel(®) Xeon(®) Gold 6130 CPU @ 2.10 GHz (6 vCPUs)
GPU	NVIDIA V100
GPU memory size	32 GB
Deep learning framework	PyTorch(2.3.1)

**Table 3:** Detection and boundary performance for landslides of different sizes.

Landslide Size	Area (m^2^)	F1-Score (%)	Boundary F1-Score (%)
Small	<1000	91.20	82.50
Medium	1000–5000	95.50	87.30
Large	>5000	96.80	90.10

**Table 4:** Performance comparison of different models for landslide detection.

Model	F1 (%)	Accuracy (%)	Precision (%)	Recall (%)	*p*-Value
CNN	85.21 ± 0.32	82.95 ± 0.41	75.65 ± 0.58	97.55 ± 0.29	*p* < 0.0001
CNN-LSTM	93.68 ± 0.15	93.60 ± 0.18	93.68 ± 0.22	93.68 ± 0.19	*p* < 0.0001
ConvLSTM	92.30 ± 0.24	92.60 ± 0.20	97.35 ± 0.15	87.70 ± 0.35	*p* < 0.0001
GRU	93.45 ± 0.18	93.47 ± 0.21	94.65 ± 0.25	92.25 ± 0.28	*p* < 0.0001
CNN3D	92.30 ± 0.28	92.32 ± 0.30	93.50 ± 0.20	91.12 ± 0.32	*p* < 0.0001
ResNet50	87.50 ± 28	85.60 ± 33	80.20 ± 45	96.30 ± 26	*p* < 0.0001
ConFormer-Net	95.35 ± 0.08	95.37 ± 0.09	96.85 ± 0.12	93.85 ± 0.14	—

**Table 5:** Comparison of computational efficiency among different models.

Model	Parameters	FLOPs (G)	Inference Time (ms/Sample)	Training Time (h/Run)	Peak GPU Memory (MB)
CNN	105 K	0.52	4.8	1.0	1280
CNN-LSTM	366 K	2.85	17.2	4.2	3050
ConvLSTM	319 K	3.56	22.5	5.6	3520
GRU	300 K	2.43	15.8	3.9	2880
CNN3D	287 K	4.18	19.8	5.1	3380
ResNet50	850 K	1.82	10.3	2.2	2150
ConFormer-Net	802 K	7.63	31.4	7.8	5420

**Table 6:** Detailed pixel-level performance of ConFormer-Net in the leave-one-region-out experiment.

Held-Out Region	F1-Score (%)	Precision (%)	Recall (%)	IoU (%)	Dice (%)	Specificity (%)	FPR (%)
Hiroshima	93.52	95.10	92.00	87.82	93.52	99.21	0.79
Indonesia	91.85	93.40	90.40	84.97	91.85	98.95	1.05
Italy	94.68	96.20	93.20	89.88	94.68	99.42	0.58
Itogon	90.10	92.00	88.30	82.03	90.10	98.72	1.28
Average	92.54	94.18	90.98	86.18	92.54	99.08	0.93

**Table 7:** Performance of ConFormer-Net under different assumed event-time settings.

Assumed Event Time	F1-Score (%)	Precision (%)	Recall (%)
Original (t = 7)	95.27	96.79	93.79
Shifted (t = 4)	94.85	96.50	93.25
Shifted (t = 5)	94.91	96.55	93.30

**Table 8:** Performance of ConFormer-Net using the first post-event acquisition and the full temporal sequence.

Input Configuration	F1-Score (%)	Precision (%)	Recall (%)
Single Post-Event (t = 7)	86.25	88.10	84.50
Full Sequence	95.27	96.79	93.79

**Table 9:** Ablation results of different modules and architectural configurations of ConFormer-Net.

Model	Params (K)	F1-Score (%)	Accuracy (%)	Precision (%)	Recall (%)
Full Model (Ours)	802	95.27	95.29	96.79	93.79
Core Module Ablation					
w/o cross-attention (concatenation)	769	95.12	95.10	96.50	93.60
w/o cross-attention (summation)	769	95.20	95.18	96.62	93.68
w/o cross-attention (gated fusion)	774	95.15	95.12	96.55	93.65
w/o temporal modeling	105	86.89	85.43	85.00	95.48
temporal: uni-LSTM	383	90.83	90.71	90.00	90.96
Spatial Component Ablation					
w/o dilated convolution (r = 1 only)	798	94.20	94.18	95.80	92.65
w/o spatial attention	798	94.85	94.82	96.15	93.58
different dilation rates (e.g., 1, 2, 8)	802	95.10	95.08	96.58	93.52
different dilation rates (e.g., 1, 3, 5)	802	95.05	95.03	96.50	93.45
Temporal Component Ablation					
w/o positional encoding	802	94.78	94.75	96.20	93.40
shorter sequence (e.g., T = 10)	802	94.45	94.40	95.90	93.02
shorter sequence (e.g., T = 5)	802	92.10	92.05	93.80	90.45
Decoder Ablation					
single-layer decoder	625	94.60	94.55	95.90	93.32
heavier decoder (U-Net-like)	1150	95.31	95.33	96.85	93.82

## Data Availability

The Sen12Landslides dataset used in this study is publicly available from the Hugging Face repository. Specifically, the harmonized Sentinel-1 ascending-orbit (S1-asc) data from Hiroshima, Indonesia, Italy, and Itogon were used. A total of 6290 samples were included in the experiments. The raw data supporting the conclusions of this article can be downloaded at https://hf-mirror.com/datasets/paulhoehn/Sen12Landslides (accessed on 6 March 2026).
